# Antinormativity notwithstanding: The normative male author and politics of queer literary interpretation

**DOI:** 10.12688/openreseurope.20787.2

**Published:** 2026-03-25

**Authors:** Slaven Crnić

**Affiliations:** 1 Faculty of Humanities and Social Sciences, University of Rijeka, Rijeka, 51000, Croatia

**Keywords:** antinormativity, literature, normative male author, queer theory, masculinity studies

## Abstract

Setting off from the recent critical turn towards habitual antinormativity in queer theory, this article reconstructs various conceptualizations, contestations and retheorizations of normativity and antinormativity in queer theory and masculinity studies. While a number of contemporary gender-focused literary scholars have shown the ways in which the uncritical adoption of antinormative presuppositions results in a reductive understanding of normativity as necessarily static and backward-looking, the issue of what a post-antinormative queer theory would look like remains open to speculation. Drawing on these studies, the article formulates a concept of the “normative male author” as an exercise in post-antinormative perspective on the connections between the politics of queer inquiry, literature and masculinity.

## Dialing back antinormativity

A fitting title for an imagined book about the reception of Eve Sedgwick’s field-defining *Between Men* might be *Between Disciplines.* Todd Reeser (2015) noted that the status of *Between Men* is indicative of a broader divide between the approaches to masculinity in social sciences and humanities. Despite the influence that Sedgwick’s theory of homosociality exerted on both fields, in social-science-based research on masculinities, in contrast to literary studies, *Between Men* is “rarely cited or discussed” ( 1 on page 28). Although *Between Men* was initially “often, if hyperbolically, described as the point of origin for queer studies” ( 2 on page 119), it seems to have been more or less eclipsed in its significance by Sedgwick’s next book, the 1990 *Epistemology of the Closet.* The year of *Epistemology*’s publication has since attained the status of *annus mirabilis* for queer theory. In just three months, readers were introduced to Sedwick’s *Epistemology* and Judith Butler’s *Gender Trouble*, two texts subsequently often lumped together as the “ur-texts of queer theory” ( 3 on page 35). Somewhat ironically, given Sedgwick’s emphatic argument in *Between Men* about the necessity to overcome the separation of homosocial- and homosexuality-focused scholarship, it seems that the book itself came to exemplify the bifurcation that increasingly separated the nascent fields of masculinity studies and queer theory.

A sketched out conventional history of queer theory would go something like this. Queer theory emerged within US academia during the 1990s, with the term itself first used in 1991 by Teresa De Lauretis ( 4 on page 55). As Sedgwick (1994) explains in *Tendencies,* etymological roots of the word “queer” connote transversality: “it comes from the Indo-European root - *twerkw*, which also yields the German *quer* (transverse), Latin *torquere* (to twist), English *athwart*” ( 5 on page viii). Up until then, the word “queer” was used, among other ways, as a term of insult, thus its theoretical uptake marked a “reappropriation of the homophobic slur” ( 6 on page 152). Heavily influenced by feminism and French poststructuralism, queer theory sought out to challenge the perceived shortcomings of gay and lesbian studies “which were often essentialist and oppositional (with gay and straight clearly differentiated)” ( 6 on page 152). Queer theory adopted and methodologically employed a key poststructuralist strategy of treating every notion of stable identity as “a cultural fantasy rather than a demonstrable fact” ( 2 on page 82). The re-appropriated homophobic slur thus came to signify an intellectual movement, artistic practices, and forms of activism that defy normative categories of stable identities (even though it is sometimes used solely as a synonym for “gay” or “lesbian”). ^2, 7, 8^ The intellectual history of the concept of “queer” is thus often summed up in the following way:

“Queer” has been deployed as an affirmative and performative term which resists becoming a fixed category and thus gives voice to those elided or marginalised by “gay” and “lesbian” studies: bisexuals, transexuals, sado-masochists, for example. It is thus the very identificatory slipperiness in the term which maintains its political potential. ( 9 on page 82)

As we can see, there is a discourse of resistance and opposition to stability and identity that flags each aspect of queer theory’s developmental narrative. This discourse of “antinormativity” crucially informs a central feature of queer theory, namely the oppositional grounds in which queer theory establishes the relationship between normativity and queerness. As Robyn Wiegman and Elizabeth A. Wilson (2015) explain, “antinormativity reflects a broad understanding that the critical force of queer inquiry lies in its capacity to undermine norms, challenge normativity, and interrupt the processes of normalization—including the norms and normativities that have been produced by queer inquiry itself” ( 10 on page 4). Antinormativity was shown to have provided queer theory with a field-consolidating impetus, a distinct theoretical profile, and a political purpose built upon casting “antinormative subjects or practices as potent figures for some alternate horizon of political possibility” ( 3 on page 27). As Wiegman and Wilson (2015) further point out:

the history of queer theorizing has been shaped by an antinormative sensibility, one that unites the multiple and at times discordant analyses that comprise the queer theoretical archive into a field-forming synthesis. We call this synthesis *queer studies*, and we read its interdisciplinary consolidation around antinormativity as its most productive field-defining rule. ( 10 on page 2)

Antinormativity also foregrounds some of queer theory’s more immediate interests in political life and decision-making *vis-à-vis* sexuality and subjectivity. As Will Stockton (2023) argues, “queer critical attention to norms and normativity has shaped discussions about LGBTQ visibility, queer subcultures, and the meaning and value of equality” ( 11 on page 67). Indebted chiefly, but not exclusively, to Michel Foucault’s work on biopower and sexuality, two of queer theory’s arguably most widely recognized concepts – heteronormativity and homonormativity – revolve centrally around the idea of the norm, with the first denoting the heterosexuality’s sociopolitical privileges stemming out of its supposedly “natural” status, and the second referring mainly to the perceived shortcomings of identity-based mainstream LGBTIQ rights movements [ 1].

However, despite its consolidating effects and insights generated, antinormativity as such became a “rhetorical and structural ‘tic’ that repeats and repeats like a mantra of authentication in queer theory, and critical analysis more generally, (…) itself prescribed, ‘expected’ – a norm in its own right” ( 14 on page 98). Consequently, antinormativity has undergone a series of criticisms in both masculinity studies and queer theory. Queer theory, in particular, has recently been quite vocal about the need to rethink or abandon the antinormative injunction that has been shown to have reduced the theoretical scope of how we think about normativity *within* queer theory. ^3, 14– 17^ While queerness has been conceptually aligned with resistance and political change, normativity was cast as something restrictive and exclusionary, leading some queer theorists to call for an anti-antinormative reassessment of “the political common sense that claims that norms ostracize, or that some of us are more intimate with their operations than others, or that ‘normative’ is a synonym for what is constricting or controlling or tyrannical” ( 10 on page 12). Of course, this renewed interest in thinking about normativity outside the framework of antinormativity should not mean a return to essentialisms or identitarianisms of any sort. Rather, it signifies the need for an exploratory reframing of anti-essentialist epistemology that Vicki Kirby (2015) outlined with the following questions:

How should we circumscribe an entity or behavior as an appropriate starting point, one that is analytically separate from another? How, for example, can we identify or foreclose what is normative if our departure point is one that already contradicts itself, one whose identity meanders all over the place and won’t “sit still”? ( 14 on page 99)

In this article, through a crisscrossed reading of the ways in which normativity has been theorized, conceptualized, contested and reaffirmed in masculinity studies and queer theory, I will explore some of the shortcomings of antinormativity and explore a potential alternative route for theorizing normativity in the context of literary studies focused on male-authored fiction. This study aims to provide *a (nti)-antinormative* or *post-antinormative* theoretical answers to the following questions: How do we proceed with a literary analysis that centers on masculinity in novels written by male authors who have exemplified paradigmatic gendered and sexual normativity? How do we define “masculine normativity” in a way that does not ascribe to it an immutable essence, yet also does not collapse it into “non-normativity”? Finally, how does an author’s normative gendered and sexualized image regulate our critical apparatus?

My aim here is not to suggest that abandoning antinormativity goes hand-in-hand with the abandonment of masculinity studies’ and queer theory’s critical legacy. In fact, as should be clear by the end of this article, my argument is that any attempt to think of male normativity as a mutable, dynamic and productive gendered and sexualized social and literary phenomenon can only appear *within* the combined frameworks of masculinity studies and queer theory. Returning to the example of *Between Men* and its shifting importance in masculinity studies and queer theory, what follows is an exercise in traversing the very space that is not only carved out but also shared *between* these two fields of knowledge.

## The crisis and the document: antinormativity and literary analysis in masculinity studies

Despite ongoing efforts to homogenize different approaches, methods and objects of study into a veritable interdisciplinary research field, contemporary masculinity studies have largely yielded a disparate body of work rather than a unified discipline in and of itself [ 2]. ^1, 18^ However, this diverse field of research and theory shares a common focus. Masculinity studies have remained focused significantly (or predominantly) on *normative* masculinities, differing in this regard from other approaches to masculinity that focus on *non-normative* subjectivities (masculinity included), most notably queer theory, with which they overlap and remain in dialogue. ^19– 21^

Masculinity studies’ conceptualizations of normative masculinity developed through a constant dialogue with feminist and queer theory, and have mirrored many of the developments and challenges associated with the general poststructuralist understanding of gender. Poststructuralist and queer critical approaches to masculinity in the last three decades have collapsed implicit and explicit biological essentialisms that have established relations of causality or equivalence between masculinity-related social phenomena and cisgendered male bodies. By pulling focus towards masculinity as a performative social construct, rather than a manifestation of one’s biological “factuality,” the scope of what could be understood as masculinity broadened, increasingly encompassing forms of masculinity that need not be, and indeed often are not, related to cisgendered male bodies. As a consequence, masculinity studies have aimed to account for the internal heterogeneity, historical relativity and cultural arbitrariness of the phenomena the very term of “masculinity” might refer to. ^1, 18, 22, 23^

In this regard, a key theoretical axiom grounding much of masculinity studies’ research has been the causal connection between masculine normativity, gender-based domination and conceptual *invisibility.* From the outset, critical approaches to normative masculinity have dealt with the effects of the social, political and historical invisibility of masculinity as a particular *gender*. ^24^ At stake here was the unmarkedness of normative masculinity’s specific and quite particular *genderness*, an invisibility that allowed it to appoint itself as the “transcendental anchor and guarantor of cultural authority” ( 21 on page 281).

While the field of masculinity studies’ inquiries into literature is still “largely unexplored in academia, especially in comparison to literary studies on women” ( 28 on page 427), over several decades, literature has been explored as a privileged depository of masculinity’s representations, values, ideals, shortcomings and crises of legitimacy, as well as potential subversions ^18, 27– 29^ [ 3]. In particular, literature is mostly conceived of as “a privileged space and epistemological medium where the manifold mechanisms of configuring ever different and divergent masculinities in the discursive condition becomes readable, knowable, and thereby also rewriteable” ( 27 on page 5-6). In this regard, since its very beginnings, literary masculinity studies tend to overwhelmingly focus on literary works as “social documents” that “reflect different cultural conceptions of masculinity” ( 28 on page 428). For instance, already at the onset of masculinity studies, James Riemer (1987) suggested that rereading American literature from a men’s studies perspective would help consolidate the field by positioning a wide range of texts—popular as well as canonical—as social documents that reflect dominant cultural ideals of masculinity ( 30 on page 289–290).

In practical terms, this means that research applying masculinities studies to literature most often aims to elucidate representations of masculinity that can be used as explanatory or even transformative frameworks for non-fictional masculinities. As Hobbs (2013) points out, “there are two linked applications of masculinity studies to literature: to consider the more private realms in which masculine identity may be formed and performed; and to isolate and examine positive examples of male protagonists who do not conform to masculine stereotypes” ( 29 on page 391). This idea that literary studies can both contribute to understanding real-life masculinities and potentially transform male readers is shared among the fields’ formative inquiries into literary studies [ 4], while contemporary masculinity studies’ inquiries into literature are still often aiming to discover alternative modalities of masculinity. ^28^

Another almost paradigmatic route taken by both early and contemporary masculinity studies in their theoretical endeavors to make masculinity visible and analyzable as a particular gender has been to focus on moments of individual, collective or historical crises. ^1, 21, 23, 34^ On the one hand, given the field’s emphasis on the importance of historical change in researching masculinities (as is evident, for example, in the concept of hegemonic masculinity), masculinity studies have yielded considerable research output focusing on historical moments of crises: “While some took certain historical moments as more crisis-filled than other ones, other scholars viewed masculinity as always, in a certain sense, in a state of crisis” ( 1 on page 20). A corollary of this idea was the poststructuralism-inspired understanding of the unstable normative male subject. As mentioned earlier, masculinity studies have remained in close contact with queer theory, and this emphasis on critical ruptures within masculinities at the individual and collective levels has formed mainly in response to the field’s adoption of queer theory’s presuppositions about the instability of the normative subject. One such rupture indicating the intrinsic instability of normative masculinity is often assumed to take place in the realm of sexuality. Normative heterosexuality, that is, seems to be critically thought of as tentatively poised on the brink of overstepping into non-normativity. Such a murky delineation between male normativity and non-normativity was aptly illustrated by Reeser (2015) on the example of the theoretically multifaceted assessment of homophobia. Whichever way it is looked at, homophobia, rather than reinforcing the strict boundary between normativity and non-normativity, suffuses the very idea of normativity with the potentiality of a rupture:

If one of the presuppositions of queer theory is that male homophobia is attempting to expel the abject queer from within, then there is necessarily something queer about or within masculinity in the first place. Or, alternately, excessive forms of masculinity may point to an instability of masculinity that contains something queer. An anti-normative gender presentation, the hypersexual man, for instance, may act the way he does because he is attempting to expel, or is responding to, an anxiety of queerness within. ( 1 on page 30)

In his oft-cited and provocatively titled article “Academic Viagra: The Rise of American Masculinity Studies” (2000), Bryce Traister offered an early critical reading of masculinity studies’ practical application of the notion of the “crisis” and its ramifications for conceptualizing normative masculinity. Although he does not use the term “antinormativity” specifically, Traister’s text was evidently and unequivocally addressing *avant la lettre* the issue of masculinity studies’ adoption and application of queer theory’s antinormative conceptual apparatus and orientation. Traister traced the effects of masculinity studies’ dependance upon and usage of poststructuralist feminist and queer scholarship. In particular, focusing mainly on masculinity studies’ adoption of Sedgwick’s notion of male homosocial desire and Judith Butler’s theory of gender performativity, Traister’s central argument was that masculinity studies have become overwhelmingly preoccupied with the idea of a masculine subject that was centrally marked by an internal crisis. In Traisters’ words,

A shared feature of these and other theoretical treatments of male identity formation and representation is that of the incoherent or paradoxical male whose fractured self is a function of identity formation. That is, such accounts proceed from a theoretical deconstruction of masculine subjectivity whose exposition is prior to the male self’s emergence within history. In other words, it does not really matter in the theoretical account which historical pressure the American male faces. Identity after Butler ultimately performs independently and in fact prior to its historical manifestation. ( 21 on page 295)

In addition to the internal crisis of subject formation, scholars also foregrounded sociopolitical crises of masculinity, especially the one purportedly triggered by the rise of feminism and gay rights activism—a crisis that attracted both academic scrutiny and extensive media attention ( 21 on page 281). Expanding upon Traister, the feminist critic Judith Kegan Gardiner (2002) noted that the discourse of “masculinity crises” rested upon a falsified image of a historical moment of gender stability “when men were men, women were women, and everyone was happy with their social roles” ( 23 on page 14).

Returning to Traister’s critical reading of American masculinity studies specifically, the bulk of his argument rests upon the effects that the narrative of the crisis ultimately has on masculinity studies’ overall project of undoing the masculine gender’s invisibility. The purported attempt to undo the tacit, invisible domination of the masculine subject is, according to Traister, that very subject’s reinstitution by other means. Specifically, the overreliance on the narrative of “masculinity-as-crisis” becomes a regulatory script of its own that blurs the politically coherent and dominant aspects of the masculine subject while overemphasizing its allegedly deep-seated anxieties. There are several consequences of the “masculinity-as-crisis” explanatory framework. On the one hand, gay masculinity, within the narrative of crisis, remains firmly “other” to normative masculinity and is seen as one of the key triggers to the crisis itself. This means that within masculinity studies, gay masculinity is precluded from being considered on equal footing as normative masculinity, which makes Traister designate the field as “heteromasculine studies.” On the other hand, the narrative of the crisis brings normative masculinity conceptually into close proximity to queerness:

That the men formerly (and still) regarded paragons of normative masculinity stand revealed as anxious failures by the crisis theory of heteromasculine historiography may provide some comfort to the less successful, the less normative, the less erect – that is, the less “masculine” – among us. ( 21 on page 292)

Crucially, however, the narrative of normative masculinity makes it almost paradoxically impossible to address this “normativity” as anything other than “non-normativity”. Crucially, however, the narrative of normative masculinity makes it almost paradoxically impossible to address this “normativity” as anything other than “non-normativity”. With the (over-)emphasizing of incoherence, anxiety and crisis as the internal truism of masculine gender performativity, Traister argues, all opposing claims are theoretically dismissed as defensive strategies of self-naturalization.

To simplify Traister’s point here, the narrative of crisis in masculinity studies does precisely what it wants to undo: it makes normative masculinity once again invisible since it reframes the issue of “normativity” within a discourse that emerges out of an attempt to demonstrate the ultimate impossibility of any “normativity” as such. As Traister points out with regard to literary studies:

Hawthorne, Poe, Melville, Emerson, Thoreau, Whitman, Howells, James, London, Norris, Hemingway, Fitzgerald, and Eliot: these former bastions of masculinist canonicity and vessels through which the patriarchal institutions of Americanist literary and cultural analysis assert themselves, are now “monumental anxieties”, queer, non-normative, tentative, unsure, and very interesting *as men.* ( 21 on page 297–298, original emphasis)

Although Traister’s critical account of masculinity studies’ crisis narrative tackles only American- and America-focused texts published by 2000, the tendency to focus on masculine crises triggered by radical moments of historical change or a general incoherence of the masculine subject (or both simultaneously) can be seen in literary studies on masculinity focusing on literature beyond the United States and published after Traister’s article. For instance, Michael Kane’s (1999) *Mapping Masculinity in English and German Literature, 1880–1930* explores male-authored canonical representations of figures such as the double or Narcissus as originating from a profound modern crisis of masculinity. Kane thus writes that “male fantasies of self-creation or self-reproduction in the 1880s [were a] realization that patriarchy itself and male patriarchal identity were in crisis” ( 35 on page 5). Alice Ferrebe’s (2005) concept of the “masculine text” (to which I will return later) also echoes the theoretical leitmotif of an internally unstable masculinity: “Masculinity, then, is an illusion. Instability is further built into the epistemological structure of that illusion itself” ( 36 on page 14). Finally, Allan Johnson’s (2017) *Masculine Identity in Modernist Literature* provides an account of and builds upon feminist scholarship on the early 20 ^th^ century crisis of masculinity to focus on the connection between modernist literary narrative voice marked by elision and the post-World War I masculine trauma of physical mutilation and castration. ^37^ My point here is not that the explanatory framework of “crises” should be abandoned altogether or that it is somehow flawed beyond repair. Quite the contrary, there is ample evidence that a focus on periods of sudden historical changes and internal incoherences yields fascinating results, as evidenced by all the studies I have just mentioned. However, Traister’s critique of potentially overusing the crisis framework seems to resonate with subsequent critical attempts to revisit some of the theoretical presuppositions of antinormative thinking about literature in queer theory. Therefore, before exploring the potential for a combined theoretical route that would allow for reframing and retaining the notion of “normativity” when approaching male-authored literature, I first turn towards the dynamic ways in which (anti) normativity appears within queer literary studies.

## Literature and antinormative politics in queer theory

With regard to queer theory, antinormativity’s field-defining injunction has more recently been traced by Heather Love (2021) back to the now defunct and, she argues, willfully forgotten field of “deviance studies.” Focusing on the stigmatization processes of social outcasts in the post-WWII period, deviance studies examined

social problems such as crime, suicide, and homosexuality in the context of poverty, migration, and tenement housing [… and figures such as] the marijuana user, the stutterer, the jazz musician, the juvenile delinquent, and the ex–mental patient ( 38 on page 23–24).

Although by no means progressive in today’s terms, deviance studies have been politically underwritten by an attempt to theorize deviance as integral to all societies, which was a radical notion in itself. This was an “‘inclusive’ view of social problems [that] emphasized the variability and inevitability of deviance” ( 38 on page 140). By the 1970s, deviance studies were superseded by identity-based approaches to sexuality that have been linked to civil rights movements, and they were thoroughly incompatible with deviance studies’ views that posited social outcasts and stigma as deterministically unavoidable facts of social life. When the nascent field of queer theory started to oppose the identity-based understanding of sexuality by leveling against it its own “dream of radical antinormativity” ( 38 on page 17), it drew from deviance studies. It did so, however, Love argues, through a peculiar reversal:

queer studies turned the descriptive study of deviance into a normative injunction to be deviant. I argue that the field of queer studies has fundamentally misunderstood the politics of deviance studies, which aimed not to disrupt social norms but instead to recognize and create space for marginal communities and practices. ( 38 on page 37)

One of the consequences of this reversal was the deepening of the rift between queer theory and social sciences: “Queer antinormativity has taken as its explicit targets heterosexuality, the family, and gender binarism; however, it is directed as much against the protocols and epistemology of the social sciences as it is against prevailing social norms” ( 38 on page 139).

Moreover, antinormativity, alongside its definitional role for queer theory, has also continually partaken in the field’s political self-image since its inception. This is evident, as Annamaria Jagose (2015) notes, in the way “early 1990s accounts of queer theory routinely emphasized its fundamental indefinability in the present and the unknowability of its future forms” ( 3 on page 33). The sense of belonging to the field of queer theory is still predicated upon one “ *always* knowing the difference between normativity and the value of being queerly set against it” ( 17 on page 55; original emphasis). Of course, this is not to propose that queer theory should somehow be either neutral towards or in favor of normativity. Quite the contrary. As Robyn Wiegman and Elizabeth A. Wilson (2015), the editors of “Queer Theory without Antinormativity”, a special issue of the feminist cultural studies journal *differences*, explain, antinormativity fulfills several important functions within the field of queer theory. First, it is definitionally central to the field, as is the opposition of queer theory to the notion of identity. It also provides thematic coherence to queer theory’s foundational canon, connecting authors and texts that came from different disciplines, such as “Leo Bersani, Judith Butler, Michel Foucault, Gayle Rubin, Eve Kosofsky Sedgwick, and Michael Warner” ( 10 on page 3). Finally, antinormativity serves as a theoretical and political meeting point between queer theory and the adjacent fields of “feminist theory, women of color feminism, and transgender studies” ( 10 on page 3).

However, what all the critical perspectives recounted above actually point towards is the way in which queer theory’s near-mandatory antagonism towards normativity inflects and distorts normativity itself as an object of queer theory’s study. In other words, there is a serious flip side to antinormativity’s formative effect on queer theory. As Wiegman (2015) notes, it positively charges equivalence it itself produces between the concept of queerness and political transformation. Consequently, through such a lens and political alignment, normativity can appear only as stasis. Wiegman’s take on this is worth quoting at length:

normativity is transformed from its status as an object of study into the figure that renders political the field’s own institutional ambitions. In this condensation, normativity is overwritten by the ahistorical presumption that it is always regressive and constraining – in short, that it is always politically bad. (…) The issue this essay raises is more simple if vexing precisely because any effort to consider normativity a complex object of study is so decisively at odds with the transgressive fictions that underwrite the field’s sovereign declarations (…) it is increasingly the case that a studied approach to the complexity of normativity as it operates across the spheres of social and psychic life is precisely what antinormativity enables the field to most actively resist. ( 17 on page 55, 66)

The issue of certain objects being preempted from appearing in queer theory due to antinormative injunctions has been raised in a number of literary studies. Preceding and arguably anticipating recent critical inquiries into the shortcomings of antinormativity, there were two important gender-focused literary studies addressing aspects of antinormativity *avant la lettre.*

In *Between Women: Friendship, Desire, and Marriage in Victorian England*, Sharon Marcus (2007) draws from Eve Sedgwick’s work on homosociality and queer theory to revisit cultural history and literary analysis of 19 ^th^ century female homosociality. Specifically, Marcus tackles the theoretical presuppositions guiding scholarship on Victorian women that have “made it difficult to conceptualize friendships between women who embodied feminine norms” ( 39 on page 12). In particular, what made friendship between normative women imperceptible in research was, Marcus argues, the operating idea that female homosociality was dominated by women’s relationships with men and, furthermore, its opposition to or reappropriation of masculinity. This was echoed in the most prominent field of research on female friendship and lesbian studies. The foundations of Marcus’ argument are strikingly similar to the subsequent critique of antinormativity in queer theory: the critical emphasis on *resistance* to heterosexuality and men in general obfuscated what Marcus set out to reconstruct, namely, the dynamics of friendship among women. Once reaffirmed in their own homosociality, rather than being viewed solely through the optics of their relationship with men, normative women could be seen as having created and maintained a vibrant, active, emotionally and physically highly-charged world of female homosociality. Importantly, Marcus intervenes in literary studies by demonstrating how Victorian marriage plots, most often read as centered around the heterosexual couple, are actually framed around female friendship [ 5].

In *Queer Dickens*, Holly Furneaux (2009) suggests that following the antisocial turn [ 6], queer theory narrowed down its epistemological scope by rejecting domesticity, family and kinship from its purview. The main issue here is again framed similarly to the problematics of antinormativity, as it was subsequently defined. Specifically, Furneaux argues that antisocial strands in queer theory have conflated heteronormativity with any notion of domesticity or kinship and proposes that queer theory should recognize “the validity and importance of a variety of conjunctions outside, and indeed, antithetical to, a central domain of queer theory as it is currently constituted: queer parenting, queer family, queer domesticity, queer tenderness, and queer happiness” ( 40 on page 12). Abandoning the antisocial framework, and pursuing, instead, the historicist argument that “there is nothing natural in the near synonymy now attributed to the familial and heteronormativity” ( 40 on page 14), Furneaux inscribes kinship and domesticity back into the queer rubric by focusing on Dickens’ literary representations of nurturing masculinity, bachelor fathers and tender male tactility.

Since the 2015 special issue of *differences*, the topic of (anti) normativity has gained significant traction in queer studies. Ben Nichols (2020) recently argued in his monograph *Same Old: Queer Theory, Literature and the Politics of Sameness* that the topic of normativity has been largely abandoned in queer theory because it was most often considered as a form of *sameness.* In a nutshell, Nichols argues that queer theory emerged from a broader poststructuralist foundational privileging of *difference*, conceived of as a site of positive progress and change, over *sameness*, conceived of as stasis and stagnation. As part of this epistemological hierarchy, queer thinking, Nichols argues, “poses values like heterogeneity, variety, multiplicity and change in opposition to a prevailing order that is imagined as seeking sameness in the forms of homogeneity, fixity, mainstreaming and conformity” ( 16 on page 3). Nichol’s approach, in his own words, “does not accord with the more prominent models available for understanding ‘sameness’ in queer scholarship and culture, which imagine it as a force of grand ontological disintegration with profound ethical implications” ( 16 on page 22). Instead, Nichols identifies politically positive inflection in modalities of queer sameness that are tied to uselessness, reproduction, normativity, and reductivity. With normativity in particular, Nichols identifies a distinct 20 ^th^ century genre of middle-brow lesbian novel, such as Radclyffe Hall’s *Well of Loneliness.* This tradition of writing has demonstrably framed lesbian relationships in distinctly ordinary, normative terms, seeking comfort in aspiring to and defending the importance of middle-class mores and normativity, which, in turn, has impacted and popularized modern lesbian identity. Thus, Nichols concludes, “even if queer theory has been staunchly anti-normative, the phenomena that one might associate precisely with the normative have none the less played a crucial role in queer history, particularly in making available certain kinds of queer identity” ( 16 on page 119).

Finally, both Kadji Amin (2017) and Michael Lucey (2019) argue that queer theory’s political self-idealization, which, as we have seen earlier, goes in tandem with antinormativity, reduces the scope of its insights by insisting on a forward-oriented, futural political progressivism. Writing about the “antinormative coalition across difference” of queer theory, Amin (2017) notes how “[i]n much queer scholarship, what binds coalition is negatively defined — it is a shared *abjection*, an *exclusion* from normativity, a common *marginalization* as deviant, a *disidentification* with hegemonic ideals, or a stance of *opposition* in relation to state power” ( 15 on page 172–173; original emphasis). The negative definition then undergoes a process of idealization through which queer theory “remains driven by a set of temporal values that orient it, almost triumphally, toward futurity” ( 15 on page 33). However, an immediate problem here is that such an antinormative definition of queerness excludes the “backward” looking subjects and objects that do not conform to this projective political utopianism. For instance, in his recent book *Someone: The Pragmatics of Misfit Sexualities, from Colette to Hervé Guibert*, Lucey (2019) takes off from Judith Butler’s framing of the concept of “queer” as turned towards “futural imaginings” and the “expanding [of] political purposes,” and notes that many experiences of non-normativity do not conform to such a politically progressive definition ( 42 on page 2). In fact, Lucey demonstrates that many writers we usually consider queer were in fact indifferent towards or resisted defining their sexuality in any terms whatsoever. Many were “more past oriented than future oriented,” conservative or reactionary ( 42 on page 2).

To summarize, what all of these studies, each in its own way, point towards is that some gendered and sexualized literary subjects can only appear in theory once we skew or abandon queer theory’s antinormative injunction in which a “properly” queer object is marked by its irrepressible resistance to normativity that is “properly” static and backward-looking. It is precisely this (auto) perception of normative masculinity that Judith Gardiner (2002) differentiated from the political utopianism of contemporary feminism. While acknowledging the “asymmetrical interdependencies of masculinity and feminism as cultural formations and academic subjects,” Gardiner contrasts the temporal dimensions of their political profiles. While feminism projects itself into an idealized futurity,

[m]asculinity is a nostalgic formation, always missing, lost, or about to be lost, its ideal form located in a past that advances with each generation in order to recede just beyond its grasp. Its myth is that effacing new forms can restore a natural, original male grounding. (…) Both fantasies risk simplifying the political — into the invisible hand of the natural or the dichotomous ally or enemy of the ethical — and both mystify collective action as too futile or too effective. Both are myths of power: masculinity of the natural congruence of male self with social privilege and feminism of a perfectly self-regulating collectivity. ( 23 on page 10–11)

The political utopianism of feminism, as described by Gardiner, seems akin to the way in which queer theory aligns itself with a futural political progressivism hinged upon, as we have seen, antinormative theoretical presuppositions that cast the very idea of normativity on the role of perpetual (political, social, subjective) stasis. Circling back to the discussion about antinormativity and its perceived theoretical and methodological dead angles (or dead ends), one question worth exploring here is the agential lens – or the lack thereof – through which normative masculinity appears as an object of literary analysis within both queer and masculinity studies. In other words, how are we to dynamize the gender-focused analysis of literary works authored by men who epitomized utmost normativity in order to go beyond the frameworks of antinormativity, “crisis” and “the social document,” but without abandoning the overall theoretical profiles of queer and masculinity studies?

The challenge here, it seems, would consist of reframing the concept of masculine normativity within the intersecting theoretical frameworks of masculinity studies and queer theory in a way that would allow it to be at least part of the multifaceted agency that queerness attains at the intersection of queer theory and literary studies. Unlike the presumed static, nostalgia-oriented profile of masculine normativity, queerness – in all its semantic capaciousness – is imbued with a sense of agency that encompasses the figure of the author, the text and the reader. Consider Noreen Giffney’s (2009) description of the meticulousness of queer classics (including Sedgwick’s and Butler’s groundbreaking works):

There is a careful attention to detail, not just to what is being said, but also to the context within which narratives unfold. Queer becomes, in these works, either a tool to decipher texts, a facilitative environment within which the reader’s relationship with a text develops or an ontological property waiting to be uncovered within the text itself. ( 43 on page 7)

My argument here is that the analysis of the gender-specific poetic stamp on texts produced by authors who themselves epitomized normativity, a stamp isomorphic to the “ontological property” of textual queerness, would require the abandonment of antinormative premises if masculine normativity is to be observed in its poetic, that is, meaning-making capacity, and not reduced to, for example, the author’s personal homophobia, machismo, latent queerness, inescapable crisis, or an archive of social patriarchal ideology. Parsing out normativity in this way to render it outside an antinormative framework seems necessary not least because

[b]y exploring the difference between a norm and the terms that often define it—domination, homogenization, exclusion, identity, or more colloquially, the familiar, the status quo, or the routine—we demonstrate the importance of the conceptual and political distinctiveness of normativity as an object of inquiry. In particular, our goal is to show that norms are more dynamic and more politically engaging than queer critique has usually allowed. ( 10 on page 2)

## (Dis)appearance of the normative male author

Thinking about *post-antinormative* queer theory proves to be a slippery terrain. Will Stockton (2023), for instance, opines that a possible way to answer Wiegman and Wilson’s question of what antinormativity-free queer theory would look like is to return to Freud’s “Three Essays on the Theory of Sexuality” that posit a general perversion at the heart of every norm since “the exhibitionism and voyeurism (nudity), sadism and masochism (penetration, orgasm) [are] present in the most ordinary of sexual encounters” ( 11 on page 87). While arguably thought-provoking, Stockton’s proposition offers a somewhat circular argument. For one, the very notion of a necessarily perverted norm sounds like an antinormative idea in and of itself and hardly a curiosity-driven revisiting of the logic of the norms. Second, Freud’s psychoanalysis, and in particular his undermining of the idea that the self exists in a coherent and stable state, has always been recognized as one of the theoretical foundations of queer theory. ^2^ A return to Freud would seem to be a decisive return to antinormativity rather than a departure from it.

With regards to the issue of masculine normativity and literature, one way to eschew the reductionist aspects of the frameworks of “social document” and “masculinity-as-crisis” would be to uncouple the normative male author and his text from the interpretative force of antinormativity, and address the issue of normativity on its own terms, rather than to analyze it with an antinormative outcome in sight. In other words, to explore the interpretative terrain that opens up once the male authorial persona is not seen as inadvertently (or otherwise) voicing either social or his own personal gendered insecurities, anxieties and failures. However, further questions immediately arise. Is there a way to view normativity *as* normativity? What can be seen once our focus is turned away from fractures and crises? How do we account for normativity once we acknowledge that it can be quite self-assured and not necessarily in crisis, although acutely aware of its others and historical change? And would not such an uncoupling run the risk of reinstating the very notion of a universalized and invisible, de-gendered “male author” that feminism and masculinity studies worked hard to bring into critical focus?

It bears pointing out that using the concept of “normative male author” to theoretically encompass a facet of masculinity that is irreducible to its presumed inner crises and anxieties triggered by a general instability of the gendered self is not inherently an attempt to essentialize anyone’s gender or sexuality. The point here is not, of course, to advocate resorting to the very concepts that queer theory has most forcefully and persuasively demonstrated as ultimately untenable: a stable and coherent gender identity conjoined with a likewise stable and coherent sexual orientation. Rather, the point is to imbue the very notion of “normativity” with some of the agential force that queerness is bestowed with in antinormative thinking about queer literature. A possible starting point, then, could be to look at the text as having been authored by a figure who was epistemologically aware of masculinity’s semantic capabilities and utilized them as parts of his creative strategies. Another step could be to explore normativity as functioning similarly to queerness as another gendered “facilitative environment within which the reader’s relationship with a text develops” ( 43 on page 7).

In contrast to literary masculinities studies’ focus on the transformative effect of its analysis on imagined individual readers, Antoine Pellerin (2016) recently proposed that one of the subfield’s real main contributions could be to make visible the ways in which canonical literature and literary epistemology have been produced, shaped and marked by their engagement with masculinity. Focusing on the ways in which masculinity studies can and should intervene in traditional and institutionalized literary studies, Pellerin concluded that:

[T]he connection between writing and masculinity is historically contingent and pragmatically constituted. Writing does not take place *ex nihilo*, but is intricately woven with a network of social, cultural and aesthetic norms which precede and exceed the writing subject. Authorial identity does not emerge on the corner of a blank page, but on *the public stage of literature* [emphasis added]. The author is *well aware* [emphasis added] that his identity is going to be perceived through the reader’s eye and inferred from the stylistic characteristics of his prose. ( 18 on page 8)

Pellerin’s suggestion that “authorial identity” takes shape “on the public stage of literature” can be partly transferred to the problematics of the “normative male author” as discussed above. Whereas Pellerin emphasizes the author’s *personal* involvement with the public stage of literature, my interest here lies in the ways in which the gendered figure of the normative male author emerges through a *collective* consensus and, furthermore, functions as a regulatory interpretative horizon or, indeed, a facilitative environment between the author, the text and the reader.

In this sense, the notion of “the normative male author” I have in mind here is conceptually akin to Alice Ferrebe’s (2005) concept of “the masculine text”. Ferrebe, through an analysis of “the white, middle-class, English, heterosexual, male, fiction-making majority” ( 36 on page 1), developed the concept of “the masculine text” in order to account for the ways in which male-authored fiction immasculated [ 7] its readers, that is, coaxed them to adopt and identify with a masculine point of view. In Ferrebe’s own words “a masculine text retains an ultimate political aim – to channel desires for traditional narrative pleasure and privilege into the acceptance of a range of masculine definitions and principles” ( 36 on page 7). A “masculine text” brings together a community of immasculated readers, male and female alike, that willingly or unwittingly interiorize “the masculine standards of self and behaviour established by a text” ( 36 on page 9). Ferrebe describes this as “textual belonging to male-authored novels” ( 36 on page 7). The word “belonging” is key here because it entails the readership as it does the author and the text. While writing recently about the recurring question of *subjectivation*, or how individual psychological, sexual and ideological identities and beliefs connect to social norms, Vicky Kirby (2015) noted: “The words *norm*, *compliance*, and *shared* all draw on a sense of belonging, a sense that a language of meaning-making through which social behaviors are interpreted is held in common” ( 14 on page 99).

Following Pellerin, Ferrebe and Kirby, we can now tentatively define the notion of “the normative male author” as a specific normative gendered and sexualized modality in which the figure of the male author belongs to the readership at large. More precisely, in this usage, the notion of “the normative male author” would apply to a gendered and sexualized presumption of normativity upheld by the “public stage of literature”. Of special interest here are the ways in which “the normative male author” both invites and preempts certain kinds of critical inquiries. Thus, the point here is not that “the normative male author” is a regulatory or prohibitive figure. On the contrary, precisely because he is normatively gendered and sexualized, he generates a lot of specific interest among biographers, specialists and critics. Whether acknowledged or not, feminist, queer and masculinity studies approaches to male-authored literature undoubtedly derive at least a part of its critical exigency from the normative framework within which a given male authorial persona takes shape. Interpretative paths exploring issues such as the portrayal of women or homophobia are immediately and rightfully taken up by contemporary literary studies as pertinent because some male authors epitomize masculine normativity. The critical importance of some gendered literary topics is thus tacitly predicated upon the normative masculine framework in which the author’s persona appears to the public. Yet, as we have seen, this normative masculine framework itself remains difficult to grasp as a viable object of study outside or beyond the frameworks that seek to redress normativity as always-already non-normative, that is, functioning purely as an illusory placeholder for a never fully reified norm. Making the normative male author visible as a consensus upheld by the public stage of literature would necessarily entail probing the ways in which this normativity both produces and limits the critical reception of his novels, including the parts produced within gender-focused scholarship such as queer theory and masculinity studies. In this sense, rather than focusing on a charted antinormative interpretative destination, it becomes possible to both tackle the masculine normativity’s definitional invisibility *and* at least partly retain the norm’s double meaning of procedural and regulatory productivity.

Finally, a post-antinormative outlook on male authorship and literature would potentially reroute some interpretative energies away from the issues of an author’s presumed gender instability or a flattened conception of the “social document,” and back onto the question of gendered poetics. In other words, although the task of de-universalizing normative male subjectivity seems as urgent as ever, the time also seems ripe to revisit the significance of male normativity for and within literary texts. The recent and multipronged critical turn towards antinormativity within queer theory warrants posing methodological and interpretative questions that would help expand the perspectives of the “social document” or “crisis” when it comes to male-authored literature. Perhaps these two tasks can go hand-in-hand, rather than in opposite directions, retracing the space carved between queer theory and masculinity studies once again.

## Ethics and consent

Ethical approval and consent were not required.

## Data Availability

No data associated with this article.

## Footnotes

^1^
As Lisa Duggan first defined, homonormativity refers to “politics that does not contest dominant heteronormative assumptions and institutions but upholds and sustains them while promising the possibility of a demobilized gay constituency and a privatized, depoliticized gay culture anchored in domesticity and consumption” ( 12 on page 179). In concrete terms, this entails “the mainstream movement’s rejection of a more radical queer politics found in early alliances with other social justice campaigns … [and] the deliberate choice to disengage with questions about economic inequality and racial justice” ( 11 on page 75). The framework of homonormativity also foregrounds Jasbir Puar’s (2007) concept of “homonationalism”. ^13^

^2^
From a historical perspective, critical approaches to masculinity, known today mostly as “masculinity studies”, emerged in the late 1980s academic circles as a response to a lack of in-depth, specialized, and pro-feminist/anti-homophobic research on male life experiences and modes of socializing, cultural and artistic representations, and history. Inspired by feminist and gay and lesbian scholarship of the second half of the 20 ^th^ century, masculinity studies have focused on the ways in which the very notion of masculinity varies between and within cultures and across history, thus supplanting earlier ideas of monolithic or universal masculinity and patriarchy with a more nuanced understanding of the power dynamics and performativity inherent to both phenomena. ^1, 18, 20, 21, 25– 27^

^3^
To this conclusion I must also add that what counts as “literary masculinity studies” is not necessarily determined solely by its thematic focus on masculinity in literature. As Alex Hobbs (2013) explained, “[l] iterary masculinity studies, like other gender studies approaches to literature before it, stems from sociological concepts. In this case, the critical framework employed by masculinity studies scholars originates in men’s studies” ( 29 on page 383). In other words, research that analyzes masculinities, but uses feminist or queer theory’s apparatuses, would not, strictly speaking, be considered “masculinity studies”, but would rather fall under the rubric of feminist or queer studies.

^4^
Among other examples, see 31, 32 and 33.

^5^
An especially interesting example in this regard is Marcus’ (2007) reading of Charles Dickens’ *Great Expectations.* In contrast to most readings that focused on Estella and Pip’s love prospects, Marcus traced the novel’s central theme as one in which “man’s desire for a woman is shaped by his identification with the desire between women” ( 39 on page 170). In this case, the crucial relationship is not the heterosexual one, but the female homosocial dyad between young Estella and her adoptive mother, the eccentric and dilapidated spinster Miss Havisham.

^6^
The antisocial turn designates a body of work in queer theory (e.g. Leo Bersani, Lee Edelman, Judith Halberstam) that ascribes political and theoretical importance to meanings and affects conventionally excluded from mainstream accounts of queerness: anti-teleology, the death drive, failure, illness, addiction etc. ( *see more* 41).

^7^
The concept of “immasculation” was first developed by the feminist literary scholar Judith Fetterley in her book *The Resisting Reader* ( 44). Using American literature as her case study, Fetterly argued that male-authored literature subdued female readers into accepting patriarchal systems of values as normal and legitimate, including pervasive misogyny. Feminist literary criticism’s role was, according to Fetterley, to teach resisting, opposing modes of reading canonical literature and thus producing transformative criticism ( 44). Ben Knights (1999) redeveloped Fetterly’s concept as “estranged masculine readings”, that is, “readings which – while reflexively conscious of the gender identities of those practising them – do not accept a hegemonic masculinity as an inescapable given” ( 32 on page 23). Knight’s opined that gender-focused lens could subvert the male reader’s own unacknowledged and erased embeddedness within a large social narrative on masculinity “so that we may learn to question our own implication in or distance from the narratives addressed to us and those we ourselves address to others” ( 32 on page 108).
