# Supplementary material for: Antinormativity notwithstanding: The normative male author and politics of queer literary interpretation
Source: Open Res Eur. 2026 Mar 25;5:240. Originally published 2025 Aug 8. [Version 2] doi: 10.12688/openreseurope.20787.2 (PMC13022559; doi:10.12688/openreseurope.20787.2)
Supplement: Peer review history

# Reviewer report: Reviewer response for version 2

**DOI:** 10.21956/openreseurope.25087.r71577

**Published:** 2026-03-26

**Authors:** Anil Pradhan

**Affiliations:** 1 Jadavpur University, Kolkata, West Bengal, India

The author has satisfactorily explained their position regarding the structure and revised the article to incorporate other suggestions. I recommend the article to be indexed.

If any, are all the source data and materials underlying the results available?

Yes

Is the work original in terms of material and argument?

Yes

Is the work clearly and cogently presented?

Yes

Is the argument persuasive and supported by evidence?

Yes

Does the research article contribute to the cultural, historical, social understanding of the field?

Yes

Does it sufficiently engage with relevant methodologies and secondary literature on the topic?

Yes

Reviewer Expertise:

queer studies, queer literature, queer theory

I confirm that I have read this submission and believe that I have an appropriate level of expertise to confirm that it is of an acceptable scientific standard.

# Reviewer report: Reviewer response for version 1

**DOI:** 10.21956/openreseurope.22491.r58033

**Published:** 2025-10-16

**Authors:** Ralph J. Poole

**Affiliations:** 1 University of Salzburg, Salzburg, Austria

The article makes an inspiring and provocative intervention into debates on antinormativity by urging queer literary studies to reconsider the critical force of normativity. Although the focal point is the “normative male author,” for me, the strength of the article lies in an appraisal of the antinormativity vs. normativity discourse within queer theory and masculinity studies, and what function literary studies takes vis-à-vis social sciences. As a literary scholar, I appreciate defending literature against the reduction to “social documents.” I also support the author’s proposed shift from predominantly looking for queer subversion in literature towards acknowledging literary works that feature seemingly more normative characters. While I may not fully embrace the conceptualization of the “normative male author” as an innovative framework of meaning-making, I follow the author’s call for considering normativity through a critical post-normativity approach as an intriguing poetic function and not as a flattened, static, pathological, and crisis-ridden failure to resistance, deviance, and subversion. The article is highly conceptual, as it should be, and succeeds in explicating a fair amount of complex theory; however, I would have liked more close readings of literary examples to illustrate their post-normative aesthetics and highlight their possible political incentive. The material basis of the argument is literature and aims at reading practices emerging from that literature, after all, not theory in and of itself.

If any, are all the source data and materials underlying the results available?

Yes

Is the work original in terms of material and argument?

Yes

Is the work clearly and cogently presented?

Yes

Is the argument persuasive and supported by evidence?

Yes

Does the research article contribute to the cultural, historical, social understanding of the field?

Yes

Does it sufficiently engage with relevant methodologies and secondary literature on the topic?

Yes

Reviewer Expertise:

American Literature and Culture, Queer Theory, Gender Studies, Popular Culture

I confirm that I have read this submission and believe that I have an appropriate level of expertise to confirm that it is of an acceptable scientific standard.

# Reviewer report: Reviewer response for version 1

**DOI:** 10.21956/openreseurope.22491.r58031

**Published:** 2025-09-12

**Authors:** Anil Pradhan

**Affiliations:** 1 Jadavpur University, Kolkata, West Bengal, India

The article does a good job in bringing together relevant and compelling theoretical contexts, arguments, and offshoots into a conversation towards conceptualizing the author’s idea of the post-antinormative politics of the normative male author. The introduction is focused and concise, providing the necessary theoretical and thematic backdrop for the core argument of the article. In the latter sections, the research is relevant and clear in terms of the core argument of the author, which helps add to the fields of both queer studies and masculinity studies in a critical, timely, and nuanced manner. However, the article reads like an introductory section to a larger project, for by the time I completed reading it, I was looking forward to find at least a few examples of the literary contexts of the core argument. The author builds an expectation of engagement with literature relevant to their critical rethinking of the field(s) and the practical demonstration of the theoretical supposition but eventually does not fulfil that very key expectation. This, for a researcher of literature like me, was a disappointment. In short, there were multiple references to and examples from literary and cultural criticism that focus on literary texts but the author, themself, do not add to that conversation in an original manner, despite the promising theoretical formulation and critical/analytical promise.

 Furthermore, the number of direct quotations can be reduced; instead, the core idea can be paraphrased to improve the critical narrative of the research referred to and incorporate into the article. E.g., the quote from Riemer can be replaced with a paraphrasing of the core idea in the quotation to make the author’s discussion and argument flow seamlessly. Additionally, block quotations, wherever necessary, should be adequately synthesized, specifically highlighting the relevance in the article’s core argument; one example is the quotation from Reeser that needs some form of commentary to make it fit into what the author is trying to say by referring to him.

 Also, sentence structure issues need to be reviewed; one example is the repeated use of a comma in the format: “… not only …, but also ….” The comma is not required here as “but” is not functioning as a coordinating conjunction in this case.

If any, are all the source data and materials underlying the results available?

Yes

Is the work original in terms of material and argument?

Yes

Is the work clearly and cogently presented?

Yes

Is the argument persuasive and supported by evidence?

Yes

Does the research article contribute to the cultural, historical, social understanding of the field?

Yes

Does it sufficiently engage with relevant methodologies and secondary literature on the topic?

Yes

Reviewer Expertise:

queer studies, queer literature, queer theory

I confirm that I have read this submission and believe that I have an appropriate level of expertise to confirm that it is of an acceptable scientific standard, however I have significant reservations, as outlined above.

# Response

**Published:** 2026-02-13

**Authors:** Slaven Crnic

**Affiliations:** Department of Cultural Studies, Faculty of Humanities and Social Sciences, University of Rijeka, Croatia

Dear Prof. Pradhan, thank you very much for your thoughtful review.

 I sincerely appreciate the time and care you invested in providing such helpful feedback. As this was my first experience working with ORE, due to a number of technical issues and misunderstandings, I have only now been able to re-upload the revised version and provide my response to your comments. I have revised the sentence structure issue and reduced the number of direct quotations in order to allow for smoother reading and more streamlined argumentation.

 Many thanks for spotlighting the two examples. With regard to the first highlighted quote (Riemer), I have replaced it with a paraphrase. I have also added several sentences that strengthen the logical connection between the second quote you singled out (Reeser) and the rest of the paragraph.

 I made many other similar interventions throughout the chapter. I completely understand that the paper seems to build some sort of expectation of engaging with fiction itself. In fact, for the longest time I have entertained the idea of incorporating a piece of literary analysis in the article. While making revisions, I even tried to tie in a close reading of a work of fiction, but in the end had to give up the idea for several reasons. For one, the necessary logistics (for lack of a better word) behind incorporating literary analysis threatened to overrun the analysis of theoretical discourse from the perspectives of both form and content. Alongside providing standalone close reading of a work of fiction, an example of careful literary analysis would entail introducing several things: the work of fiction, the author, extant critical approaches to the work itself, a reconstruction of the plot, etc. All this heavy lifting would then inflate the word count to unwieldy numbers, well beyond reasonable and acceptable publication limits for a single article.

 One option would be to cut some of theoretical reflections out, but I found this procedure causing a lot of imbalance in the text and harming the intricacy of the way in which the article already crosshatched many theoretical strands of thinking about masculinity and literature.

 The choice to have this many theoretical writings in a single article came from a deliberate decision to illustrate the scope and the stakes inherent to the critical reassessment of antinormativity and, finally, add a conceptual argument of my own to the ongoing discussions. With all that said and done, I like to think of the article as a meta-theoretical piece that is focused on theory (or theories) as its main object of inquiry. With that in mind, I have ultimately decided to preserve the internal coherence of the article and argumentation that leads up to my argument about the theoretical perspective of the “normative male author” as a possible re-orientating figure for the contemporary discussions on antinormativity in queer theory and masculinity studies alike. The practical application of this conceptual framework would require a separate piece altogether, first and foremost to do justice to its object of inquiry which would, in that case, be fiction rather than theoretical writings. Once again, many thanks for taking the time to read my text.

# Response

**Published:** 2026-03-25

**Authors:** Anil Pradhan

**Affiliations:** English, Jadavpur University, Kolkata, West Bengal, India

Hi Slaven Thank you for the detailed response and clarification. I understand the limitations and appreciate the honesty. As such, I think there isn't much scope to add literary insights to the article in the present form. Thank you for revising the article per the other suggestions. I have approved your article. All the best.

 Anil
